# Risk factors for the need of hip arthroscopy following periacetabular osteotomy

**DOI:** 10.1093/jhps/hnv053

**Published:** 2015-08-27

**Authors:** Charlotte Hartig-Andreasen, Anders Troelsen, Theis M. Thillemann, John Gelineck, Kjeld Søballe

**Affiliations:** 1. Orthopaedic Research Unit, Aarhus University Hospital, Tage-Hansens Gade 2, Building 10A, DK-8000 Aarhus C, Denmark,; 2. Department of Orthopaedics, Clinical and Orthopeadic Research Hvidovre, Copenhagen University Hospital, Kettegård Allé 30, DK-2650 Hvidovre, Denmark,; 3. Department of Orthopaedics, Aarhus University Hospital, Tage-Hansens Gade 2, DK-8000 Aarhus C, Denmark and; 4. Department of Radiology, Aarhus University Hospital, Nørrebrogade 44, DK-8000 Aarhus C, Denmark

## Abstract

Despite the frequency of labral tears in symptomatic developmental dysplasia of the hip, no consensus exists regarding the treatment of coexisting dysplasia of the hip and tearing of the acetabular labrum. The purpose of this prospective, MR arthrography (MRA) based 2-year follow-up study was to identify risk factors predicting the need for a hip arthroscopy (HA) after periacetabular osteotomy (PAO). Ninety-nine patients (104 hips) scheduled for PAO were evaluated preoperatively and at 2-year follow-up. MRA was performed in all patients prior to PAO. At follow-up, patients were divided into a non-arthroscopy and arthroscopy group. The two groups were compared clinical and radiological, and risk factors for HA after PAO were calculated. Patient reported outcome measures (WOMAC, Oxford Hip and SF36) were filled out before PAO and at follow-up. Ninety-five hips (91.3%) were evaluated. Twenty-six hips (27%) required an arthroscopy within 2 years of the PAO. Risk factors were preoperative borderline dysplasia, acetabular retroversion and complete labral detachment. Labral tearing, degeneration or hypertrophy did not negatively affect the outcome of PAO. Patients not requiring an arthroscopy had a statistically significant better outcome measured by patients reported outcome measures. After PAO, 27% of the hips needed intra-articular assessment. Conventional radiographs and MRA analysis can be used to identify predictors for patients requiring HA after PAO. At 2-year follow-up, the clinical outcome improved in all patients. However, those patients who had no need of a HA after their PAO had superior results.

## INTRODUCTION

The Bernese periacetabular osteotomy (PAO) has become the preferred joint preserving treatment for symptomatic developmental dysplasia of the hip (DDH) [1]. Dorrell and Caterall [2] were among the first to report on the relationship between dysplastic osseous abnormalities and labrum pathology. Since then literature describing how the osseous abnormalities and the resulting pathological joint biomechanics in developmental DDH may frequently lead to damage of the acetabular labrum has been evolving [3–6]. Recently, Ross et al. found only 5 normal labrums in 73 dysplastic hips [7].

Despite the frequency of labral tears in symptomatic DDH and the increasing literature concerning labral pathology, no consensus exists regarding the treatment strategy for DDH with coexisting acetabular labral tear. Tearing of the labrum is recognized being involved in joint degeneration and this may untreated lead to osteoarthritis. This has lead to new concepts and treatment strategies regarding the treatment of labral tearing in DHH. Open arthrotomy during PAO was the first means of addressing intraarticular pathology during PAO surgery [8]. Later hip arthroscopy assisted PAO was introduced to assess and address any present intraarticular pathology [9]. There is no evidence that intraarticular assessment, open or arthroscopic, is superior to not assessing the joint during PAO. However, hip arthroscopy alone without addressing the bony abnormalities in DDH is in general not recommended, and studies have showed failure in DDH hips undergoing hip arthroscopy with debridement of the labrum [10], and resulted in high reoperation rates comparing mild DDH hips with normal hips [11]. Studies reporting the outcome of PAO performed without simultaneous assessment of the joint have shown high hip joint survival rates [12, 13]. However, femoroacetabular impingement after PAO has been observed with poor outcome, and some patients will require a subsequent hip arthroscopy, and it would be valuable to identify predictors for hip arthroscopy as well as assessment of the results after hip arthroscopy in PAO patients.

The purpose of this prospective, MR arthrography (MRA) based 2-year follow-up study was to identify risk factors predicting the frequency of the need for a hip arthroscopy after PAO, and finally to compare clinical and radiographic outcomes between patients require a subsequent arthroscopy and patients not requiring arthroscopy after PAO.

## MATERIAL AND METHODS

Ninety-nine patients (104 hips) consecutively scheduled for PAO due to DDH were enrolled in the study. Patients were included from January 2010 to August 2011 and all surgeries were performed or assisted by the senior author in Aarhus, Denmark. Five patients were excluded from the study, because of multiple complaints from several joints and thus, were not considered being representative for this PAO cohort. Four patients failed to show up at 2-year follow-up. Hence, the study group consisted of 90 patients (95 hips, 79 females, 52 right hips). Mean age of the patients at the time of PAO surgery was 34.1 years (range 14.5–58.9 years). Before PAO eight hips had a hip arthroscopy (Table I) and one patient had had a combined femoral and pelvic osteotomy. Twenty-three patients underwent PAO surgery on the opposite hip within the 2-year study period, and three patients had screws removed following PAO. One complication among the 95 was observed: an obturator nerve affection resulting in pain and paralysis of the adductor muscles. Another hip developed osteoarthritis. Beside that no intra- or postoperative complications was observed. Bilateral dysplasia was seen in 78% of the patients. Indication for PAO were persisting hip pain, a center edge angle of Wiberg [14] <25^o^, pelvic bone maturity, internal rotation >15^o^, hip flexion <110^o ^and Tönnis grade of osteoarthritis 0 or 1. The minimally invasive transsartorial approach was used in all cases [15]. Preoperatively and at 2-year follow-up, the clinical and radiographic outcome were evaluated. Follow-up was done primarily by one investigator (CHA), except for four patients seen by the senior author (KSO). For data analysis, the patients were divided into an arthroscopy group if a hip arthroscopy was required within the 2-year follow-up period and a non-arthroscopy group.

**Table I.** Description of the eight hips undergoing hip arthroscopy (HA) prior to the PAO

Hip	Time from HA to PAO	HA findings	HA procedures	HA after PAO
12^a^	NA	NA	NA, but no effect of surgery	No
13	1 year	Torn labrum	Labrum resection, short term effect	No
15^a^	NA	NA	NA, but no effect of surgery	No
38^a^	NA	Labrum tear	Reinsertion of labrum	No
45	3 years	Intact labrum, cartilage pieces	Removal of several cartilage pieces	No
46	6 years	Thin cartilage, loose pieces of cartilage, labrum tear	Resection of the damaged parts	Yes
49	2.5 years	Labrum a little frayed	Resection of the frayed part of the labrum	Yes
59	4 years	Labrum-cartilage separation, hypertrophic labrum, pincer, CAM	Rimtrim, labrum reinsertion, cheilectomy	Yes

^a^Information from patient, journal records not available.

HA findings, Findings during hip arthroscopy; HA procedures, Procedures performed during hip arthroscopy.

### Clinical evaluation

At 2-year follow-up, patients were interviewed regarding continued mechanical symptoms (clicking, locking and instability) from the hip joint, dysesthesia of the dermatome innervated by the lateral femoral cutaneous nerve, any kind of surgical and non-surgical treatment since the PAO. Signs of trochanteric bursitis, internal and external snapping hip were noted. Any leg length discrepancy and range of motion was measured. Impingement test and FABER test [16] were performed; however, in three hips, the tests were left out due to recent hip arthroscopy, and in two hips due to severe hip pain. Preoperatively and at 2-year follow-up, patients were requested to fill out the Western Ontario and McMaster Universities Osteoarthritis index (WOMAC) [17] , the Oxford hip score (OHS)[18] and the general health questionnaire short form 36, version 1 [19]. Each subscale of the WOMAC score was calculated. To enhance the comparability with other studies, the summarized WOMAC total score were normalized with 100 indicating the best possible score. The OHS score was given as a total score with 48 indicating the best possible score. From the SF36 data, the physical and mental component scores were subsequently calculated. Five patients failed to fill out the questionnaires preoperatively, hence only 90 hips were evaluated by questionnaires.

### Radiographic evaluation

**Fig. 1.**
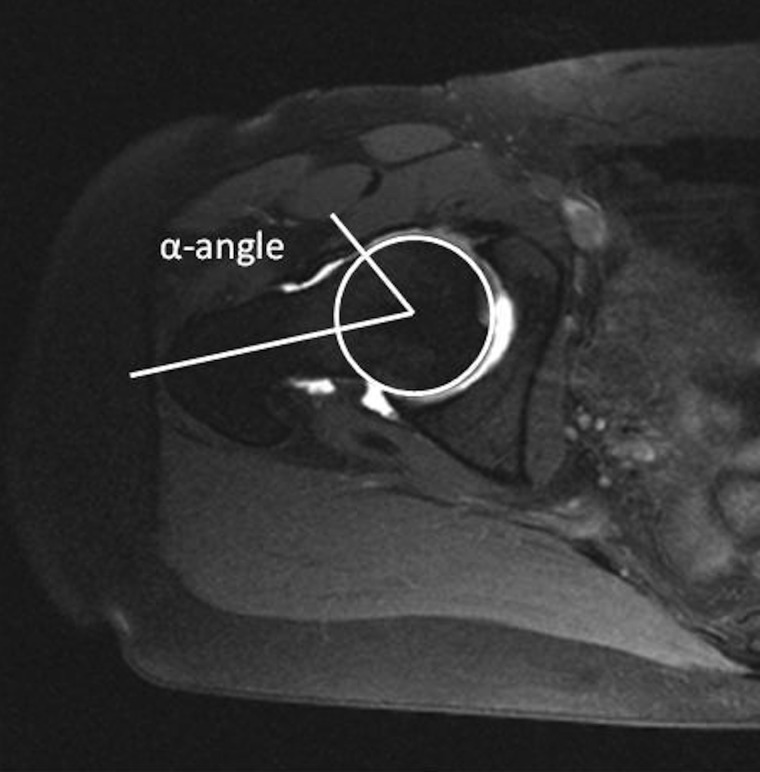
MRA measurement of the α-angle of Notzli on the oblique plane. After identification of the center of the femoral head, a line along the middle of the femoral neck and a line from the center to the point where the femoral head-neck junction ‘left’ the best fitted circle of the femoral head make up the α-angle.

Conventional standing pelvic radiographs recorded preoperatively and at 2-year follow-up were analyzed. One investigator (CHA) assessed the following radiographic parameters: the center edge (CE) angle of Wiberg [14], the acetabular index (AI) angle [20], the presence of an os acetabuli [21], the Tönnis grade of osteoarthritis [20] and signs of retroversion (cross over sign) [21]. Hips were characterized dysplastic if the CE angle was between <25^o^. AI angles were considered normal if within 0^o^–10^o^. For the hip arthroscopy group, CE angles and AI angles after PAO were analyzed at the postoperative supine radiographs, since the arthroscopy may have changed the angles. Using supine exposures were justified by an earlier study showing no significant changes in these two angles when repositioning from the supine to the weight-bearing position [22]. The acetabulum was considered retroverted if the crossover sign [21, 23, 24] was present prior to PAO. All patients had a magnetic resonance arthrography (MRA) performed. The MRA were performed with a 1.5 Tesla Scanner (Siemens Magnetom Symphony) preceded by guided injection of 10 ml of diluted gadolinium contrast medium (Gd-DTPA, 2 mmol l^−1^) into the hip joint. The MRA was assessed for labral pathology in terms of degeneration, hypertrophic changes, tears and paralabral cysts. Labral lesions were graded according to the Czerny grading [25]. Czerny stages the labrum into groups according to shape, homogeneity and attachment to the acetabular rim. Cystic changes in the femoral head or in the acetabulum were noted. The α-angle was measured on oblique axial MRA images (Fig. 1) [26]. An α-angle ≥55^o^ was considered pathological. One senior radiologist (J.G.) performed all intraarticular injections and analysis of MRA scans. Measurement of the α-angles was also performed by the C.H.A. In five hips, the α-angle could not be assessed due to imprecise oblique MRA images. Intra- and interobserver variability of the α-angle measurement was assessed by the C.H.A and the senior radiologist by doing rereadings of the MRA scan separated by 4 weeks. The mean of difference for intraobserver variability was 0.48^o^ (SD ± 1.90^o^). The 95% limits of agreement (LOA) were −3.31^o^ to 4.27^o^, and for the interobserver variability the mean difference was 1.52^o ^(SD ± 3.14^o^), 95% LOA was −4.76^o^ to 7.80^o^.

### Indication for hip arthroscopy

All patients with continuous symptoms in this study were primarily referred to the Sports Traumatology unit at Aarhus University Hospital, Denmark and evaluated by two experts in hip arthroscopy. Relevant patient history, continuous groin pain after PAO, a positive impingement or Faber test were indications for hip arthroscopy. Labral pathology diagnosed on MRA supported the diagnosis and indication. All patients referred in this study underwent hip arthroscopy.

### Statistical analysis

Depending on distribution data was presented as means with 95% confidence intervals, or as medians with interquartile ranges. Odds ratios for hip arthroscopy were calculated using logistic regression. Non-parametric variables were evaluated with Wilcoxson sign rank test or Fisher’s exact test. Intra- and interobserver variability was assessed using the Bland–Altman approach [27–29], and data was presented as means of the difference with SD and 95% LOA.

## RESULTS

Twenty-six of 95 hips (27%) had a hip arthroscopy within 2 years after PAO. One of these hips was converted to total hip arthroplasty two months after hip arthroscopy (7 months after PAO) (Table II).

**Table II.** Description of the 26 hips undergoing hip arthroscopy (HA) after PAO

Hip	Time from PAO to HA	MRA labrum diagnosis	MRA α-angle	HA findings	HA procedures
9	9 months	Czerny 3A	46^o^	Labrum damage anteriorly, mildly hypertrophic, pincer, CAM^a^	Rimtrim, labrum reinsertion, cheilectomy
10^b^	21 months	Czerny 3A, degeneration	55^o^	Frayed labrum, pincer, minor CAM	Rimtrim, labrum reinsertion, minor cheilectomy
22	13 months	Czerny 1A, degeneration	51^o^	Labrum-cartilage separation, pincer, CAM, loose cartilage	Rimtrim, labrum reinsertion, cheilectomy, mircrofracture treatment
26	6 months	Czerny 2B	53^o^	Labrum-cartilage separation, pincer, minor CAM	Rimtrim, labrum reinsertion, minor cheilectomy
31	16 months	Czerny 3A, degeneration	44^o^	Labrum-cartilage separation, pincer, minor CAM, area with osteoarthritis	Rimtrim, minor cheilectomy
33^b^	15 months	Czerny 3A, degeneration	58^o^	Labrum lesion with minor impact on the cartilage , pincer, bump on collum	Rimtrim, labrum reinsertion, minor cheilectomy
40	7 months	Czerny 3B, degeneration	44^o^	Labrum-cartilage separation, frayed labrum, pincer, CAM	Rimtrim, labrum reinsertion, cheilectomy
46	4 months	No tears, mild hypertrophy	43^o^	Labrum-cartilage separation, synovitis, pincer, CAM	Rimtrim, labrum reinsertion, cheilectomy
49	15 months	Czerny 3A	44^o^	Minor labrum-cartilage resection, minor pincer, CAM	Minor rimtrim, cheilectomy
50	12 months	Czerny 3A, degeneration	48^o^	Labrum-cartilage separation, pincer, CAM	Rimtrim, labrum reinsertion, cheilectomy
52	8 months	Czerny 3A, degeneration	69^o^	Labrum-cartilage separation, pincer, CAM	Rimtrim, labrum reinsertion, cheilectomy
54^b^	7 months	Czerny 3A, degeneration	58^o^	Voluminous labrum, labrum- cartilage separation, minor CAM	Labrum reinsertion, minor cheilectomy
58	24 months	Czerny 3A	49^o^	Lesion of the cartilage at acetabulum and femur. Labrum attached.	Minor rimtrim, cheilectomy, synovectomy
59	5 months	No tears, crushed and degeneration	49^o^	Osteoarthritis acetabulum and caput femoris, labrum attached to the rim	Synovectomy
60	12 months	Czerny 2A	48^o^	Labrum-cartilage separation, minor pincer, minor CAM	Rimtrim, labrum reinsertion, cheilectomy
64	11 months	Czerny 3A, degeneration	42^o^	Labrum-cartilage separation, pincer, minor CAM	Rimtrim, labrum reinsertion, minor cheilectomy
65	11 months	Czerny 3A	37^o^	Labrum not described, pincer minor CAM	Rimtrim, labrum reinsertion, cheilectomy
67	7 months	Czerny 3B, degeneration, hypertrophy	61^o^	Labrum-cartilage separation, mild osteoarthritis, pincer, CAM	Rimtrim, labrum reinsertion, cheilectomy
71^b^	18 months	Czerny 3B	66^o^	Degeneration of labrum, no tears, CAM osteoarthritis at acetabulum and femur	Cheilectomy
72	5 months	Czerny 3A	72^o^	Labrum-cartilage separation and influence of the cartilage, pincer, CAM	Rimtrim, labrum reinsertion, cheilectomy
75^b^	7 months	Czerny 3A	57^o^	Not available	According to the patient ‘some bone work’. No effect.
81	11 months	Czerny 3B	54^o^		Rimtrim, labrum reinsertion, cheilectomy
82	9 months	Czerny 3A, degeneration	NA		Rimtrim, labrum reinsertion, cheilectomy
85^b^	4 months	Normal	50^o^	Labrum tear	Rimtrim, labrum reinsertion, cheilectomy
95	11 months	Czerny 3A	45^o^	Labrum tear	Labrum reinsertion
100	16 months	Czerny 3A	56	Labrum tear, minimal pincer	Minimal rimtrim, labrum reinsertion

^a^CAM term for the exostose on the femoral head–neck junction.

^b^(10) Repeat arthroscopy 11 months after first HA: refixation of labrum, minor rimtrim of the acetabulum and extended cheilectomy on femur. (33) Repeat arthroscopy 14 months after first HA: labrum healed, acetabular cartilage with wave-sign, minor rimtrim, minor cheilectomy, screw removal. (54) Repeat arthroscopy 8 months after first HA: labrum healed, minor pincer removed, minor cheilectomy, psoastenotomy. (71) Hip arthroplasty 6 months after HA. (75) Repeat arthroscopy 3 months after first HA. Labrum attached but anterior lesion. Pincer and minor CAM. Detachment of the labrum, rimtrim, reinsertion of labrum and minor cheilectomy. Psoastenotomy. (85) Repeat arthroscopy 11 months after first HA. (95) Repeat HA 9 months after HA: cheilectomy.

No significant differences in sex were found between the non-arthroscopy and arthroscopy group Significant adjusted predictors of need for hip arthroscopy after PAO were (i) mild dysplasia (OR 2.92); (ii) presence of the cross-over sign on preoperative radiographs (OR 3.30) and (iii) labrum detachment (Table III). The MRA analysis of the acetabular labrum revealed only six labrum (five in the non-arthroscopy group) without any signs of degeneration, hypertrophy or pathology according to the Czerny grading. No significant differences in labral pathology were found between the non-arthroscopy and arthroscopy group (Table IV). For the arthroscopy group, the median preoperative CE angle for was 20^o^ (range 11^o^–24^o^) and the AI angle was 14^o^ (range 8^o^–21^o^). The postoperative CE angle and the AI angle was 34^o ^(range 17^o^–46^o^) and 3^o^ (range −8^o ^to 16^o^), respectively (Table V). At follow-up, the median CE angle and AI angle was 34^o ^(range 25^o^–40^o^) and 1^o^ (range −8^o ^to 16^o^), respectively. For the hip arthroscopy group, both the CE angle and AI angle changed significantly after arthroscopy (Table VI). The median α-angle for the arthroscopy group was 50^o^ (range 37^o^–72.^o^), with no significant difference between the non-arthroscopy and arthroscopy groups. About 18 of 95 hips were retroverted preoperatively, and four hips at 2-year follow-up, all in the non-arthroscopy group. Clinical testing after PAO for signs of impingement, trochanteric bursitis or persisting dysplasia revealed no significant difference between groups (Table VII).

**Table III.** Odds ratios for predictors of clinical failure in terms of hip arthroscopy (*n* = 95^a^)

Parameter	OR (95% CI)	*P* value	Adjusted OR (95% CI)^b^	*P* value
Borderline dysplasia (CE-angle ≥20° to <25°)	2.82 (1.11–7.14)	*0.029*	2.92 (1.13–7.52)	*0.026*
Postoperative AI angle <0° or >10°	2.08 (0.77–5.65)	0.151	2.48 (0.85–7.15)	0.093
Preoperatively cross over sign present	3.52 (1.21–10.28)	*0.021*	3.30 (1.09–9.95)	*0.035*
α-angle ≥55^o^	1.47 (0.55–3.92)	0.442	1.43 (0.52–3.94)	0.493
Labrum detachment	2.28 (0.81–6.38)	0.118	3.83 (1.18–12.44)	*0.025*
Labrum degeneration	0.73 (0.30–1.79)	0.486	0.88 (0.34–2.27)	0.787
Labrum hypertrophy	3.62 (0.77–17.01)	0.103	3.36 (0.69–16.42)	0.134
Presence of paralabral cyst	2.31 (0.61–8.72)	0.215	2.06 (0.53–7.98)	0.295

^a^Five hips excluded from the analyses involving the α-angle.

^b^Adjusted for age (≤35 years)and borderline dysplasia.

**Table IV.** Magnetic resonance arthrography characteristics (results for all hips and in groups, number of hips)

Parameter	All hips	Arthroscopy group (*n* = 26)	Non-arthroscopy group (*n* = 69)
Degeneration of the labrum			
Yes	53	13	40
No	42	13	29
Hypertrophied labrum			
Yes	18	2	16
No	77	24	53
Paralabral cyst			
Yes	17	3	16
No	76	23	53
Classification of labrum pathology			
0	12	3	9
1A	3	1	2
1B	2	1	2
2A	14	1	13
2B	3	1	2
3A	44	16	28
3B	17	4	13

^a^No significant differences in labral pathology were found between the nonarthroscopy and arthroscopy group.

**Table V.** Radiographic characteristics before and after PAO

Parameter	Nonarthroscopy group (*n* = 69)	Arthroscopy group (*n* = 26)	*P* value
Before PAO			
Center-edge angle			
Median (interquartile range)	17° (13° to 20°)	20° (17° to 21°)	0.055
Range	−10° to 24°	11° to 24°	
Acetabular index angle			
Median (interquartile range)	15° (12° to 20°)	14° (12° to 18°)	0.222
Range	0° to 33°	8° to 21°	
After PAO			
Center-edge angle			
Median (interquartile range)	34° (29° to 36°)	34° (32° to 37°)	0.317
Range	17° to 40°	25° to 46°	
Acetabular index angle			
Median (interquartile range)	3° (1° to 6°)	1^o^ (−1° to 3°)	0.010
Range	−3° to 16°	−8° to 16°	
Crossover sign before PAO^a^			
Before PAO	9	9	0.036
At 2-year followup	4	0	0.572

^a^Crossover sign before arthroscopy were not possible to evaluate, since postoperative radiographs after PAO were supine taken.

**Table VI.** Description of the changes in CE-angle in the arthroscopy group (*n* = 25^a^)

Parameter	Before PAO	Before arthroscopy	After arthroscopy	*P *value^b^
Center-edge angle				
Median (interquartile range)	20° (17° to 21°)	34° (32° to 37°)	32° (29° to 36°)	0.002
Range	11° to 24°	25° to 46°	22° to 40°	
Acetabular index angle				
Median (interquartile range)	14° (12° to 18°)	1° (−1° to 3°)	4° (0° to 5°)	<0.001
Range	8° to 21°	−8^v^ to 16°	−4° to 16°	

^a^One hip had only pre-arthroscopy radiographs and was left out for this analysis.

^b^Statistically significant difference between CE angles and AI angles before and after arthroscopy.

**Table VII.** Clinical findings at 2-year follow-up

Parameter	Arthroscopy group	Nonarthroscopy group	*P *value
Positive impingement	8	25	0.796
Positive FABER	5	13	0.544
Positive impingement and FABER	3	12	0.753
Trochanteric bursitis	4	11	1.000
Persisting dysesthesia	16	35	0.367

**Fig. 2.**
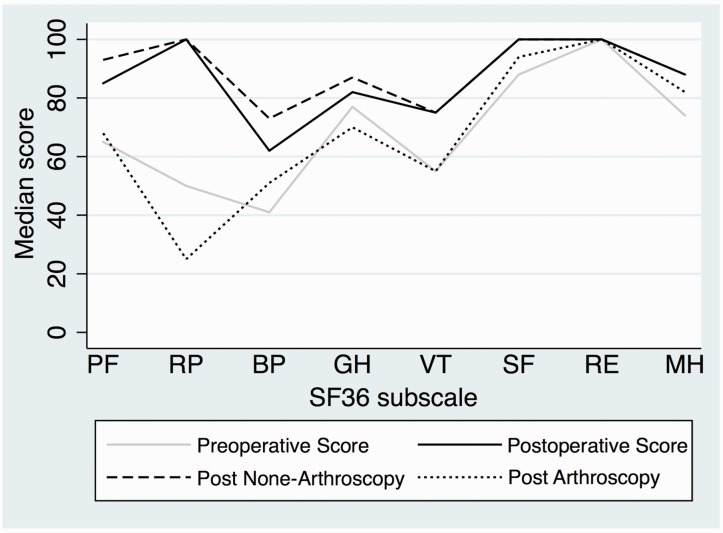
Changes in SF36 subscale parameters for 90 patients before PAO and at 2-year follow-up after PAO. The postoperative subscale parameters are also illustrated separately for the nonarthroscopy group (dash) and the arthroscopy group (dot). SF36 consist of eight subscales with health-related parameters: physical functioning (PF), role-physical (RP), bodily pain (BP), general health (GH), vitality (VT), social functioning (SF), role emotional (RE) and mental health (MH).

For both groups, the median normalized WOMAC total score increased from 66 (range 3–100) preoperatively to 89 (range 25–100) postoperatively and the median OHS increased from 28 (range 8–47) to 43 (range 12–48). The overall SF36 physical and mental component scores increased from 38 (range 16–55) to 48 (range 18–60) and from 54 (range 29–69) to 58 (range 27–78), respectively, (Table VIII). Improvements between the preoperative and 2-year follow-up assessment were observed in 7 of 8 subscales of the SF36 (Fig. 2). The total WOMAC score, the OHS and the physical component score of the SF36 differed statistically significant with superior results in the non-arthroscopy group compared to the arthroscopy group (*P* ≤ 0.001–0.013) (Table VIII). The preoperative scores for all patient reported outcome measures did not show any statistically significant differences between the arthroscopy and non-arthroscopy groups (*P* 0.067–0.810).

**Table VIII.** Patient reported outcome measures for arthroscopy and nonarthroscopy group (*n* = 90 hips)

Parameter	Preoperative^a^ All (*n* = 90)	Postoperative All (*n* = 90)	Postoperative Arthroscopy (*n* = 26)	Postoperative nonarthroscopy (*n* = 64)	*P* value
WOMAC^b^					
Pain					
Median (interquartile range)	7 (5–10)	2 (0–6)	5 (1–9)	1 (0–4)	<0.001
Range	0–20	0–14	0–14	0–12	0.013
Stiff					
Median (interquartile range)	3 (2–4)	2 (0–2)	2 (1–3)	1 (0–2)	0.001
Range	0–8	0–7	0–7	0–6	<0.001
Physical function					
Median (interquartile range)	19 (11–29)	4 (0–11)	10 (3–24)	2 (0–7)	
Range	0–61	0–49	0–46	0–49	<0.001
Total scores					
Median (interquartile range)	30 (17–41)	8 (1–22)	18 (8–36)	5 (1–14)	
Range	1–89	0–67	0–67	0–66	
Normalized					
Median (interquartile range)	66 (56–78)	89 (73–98)	78 (62–89)	91 (82–100)	
Range	3–100	25–100	25–100	33–100	
Oxford hip score^c^					
Total score (0–48)					
Median (interquartile range)	28 (23–33)	43 (35–47)	36 (28–42)	43 (37–47)	<0.001
Range	8–47	12–48	12–48	19–48	
SF36					
Physical component score (0–100)					
Median (interquartile range)	38 (33–44)	48 (38–55)	39 (32–48)	51 (44–56)	<0.001
Range	16–55	18–60	18–58	27–60	
Mental component score (0–100)					
Median (interquartile range)	54 (43–61)	58 (52–61)	56 (50–61)	58 (52–61)	0.243
Range	29–69	27–78	27–78	35–66	

^a^The preoperative scores did not show any statistically significant differences between the arthroscopy and nonarthroscopy groups (*P *values 0.067–0.810).

^b^Raw scores with ‘0’ indicating best results. Normalized score with ‘100’ indicating best result.

^c^Score with 48 indicating best results.

## DISCUSSION

This study identifies radiographic predictors for the need of a hip arthroscopy 2 years after PAO. At 2-year follow-up, a statistical difference in patient reported outcome measures between the non-arthroscopy and arthroscopy group were found.

In mild dysplasia, only little reorientation is possible before overcorrection may occur, which could be the reason for the finding of a CE angle of 20^o^–25^o^ being a significant predictor for subsequent arthroscopy. However, in this study a negative AI angle is not a significant factor similar to earlier findings reported by Steppacher [30]. Though, femoroacetabular impingement after PAO for hip dysplasia is well known [8].This could advocate for a thorough intraoperative assessment of femoroacetabular impingement. By restricting simultaneously intraarticular surgery only to patients with mild dysplasia the majority of the patients will avoid over-treatment and thereby the risk of unnecessary complications. However, a recent study of 26 patients with mild dysplasia undergoing arthroscopic treatment alone, demonstrates at 2-year follow-up significant improvement in patient reported outcome measures and VAS score [31].

In the present study, the non-arthroscopy group and the arthroscopy group showed improved WOMAC, OXFORD and the SF36 scores at 2-year follow-up. For all scores, the results for the non-arthroscopy group are superior to that in the arthroscopy group. However, when dichotomizing the WOMAC pain score into a no or low pain score group (WOMAC pain score <10) or a high pain score group (WOMAC pain score of 10 or more), 90% of patients (21 in the arthroscopy group and 60 patients in the non-arthroscopy group) had no or low pain. No statistical difference was found between groups. Finally, the intention to treat analysis of this study evaluated outcome at 2 years after PAO. The statistical difference found in patient reported outcome measures may be a result of the arthroscopy group only having mean 11.5 months (range 0–20.5 months) of follow-up between hip arthroscopy and the 2-year follow-up after PAO. Thus longer follow-up is needed to evaluate the final clinical result after delayed hip arthroscopy. However, excluding seven patients (seven hips) who had a hip arthroscopy within 6 months from 2-year follow-up did not change association. By including patients who previously underwent hip arthroscopy, the result of this study could possibly be biased. However, since this is a prospective cohort study illustrating daily clinical practice, these patients were not excluded.

Hip arthroscopy is offered to the patient by two experts at the Sports Traumatology unit, if the clinical findings suggest intraarticular pathology. However, the decision to offer a hip arthroscopy is multifactorial and it is difficult to apply narrow clinical indications regarding this end-point. MRA is considered the gold standard in imaging labral tears, but hip arthroscopy gives a direct view of the intra-articular status including any chondral damage. This means relying only on MRA findings and clinical tests, chondral damage may be overlooked. A study by Mechlenburg et al. showed unchanged status of cartilage thickness 2½ years after PAO assessed on MRI preoperatively and at follow-up indicating that osteoarthritis do not progress during follow-up even in the presence of a labral tear [32]. Fujii et al. [33] however, did find that advanced intra-articular lesions at the time of hip joint preserving surgery were a significant risk factor for high rate postoperative progression of osteoarthritis of the hip joint. Czerny’s classification of labral tears was in an earlier study found not to be prognostic for outcome [34] and Matheney et al. [35] found that a labral tear did not predict failure in terms of conversion to a THA after PAO. However, in this study a complete detachment of the acetabular labrum from the rim seen at MRA (Czerny 3A or 3B lesions), is a predictor for the need of a hip arthroscopy after PAO.

An interesting finding in this present study was that almost all patients with dysplasia had MRA verified pathology of the labrum and it is interesting to note that a great deal of the patients had a positive effect of PAO alone without addressing the labrum. We believe that redirection of the acetabulum results in significantly reduced load on the labrum which probably explains the good clinical result even when lesions of the labrum are present.

In conclusion, 27% (26 hips out of 95) of the hips underwent hip arthroscopy within the first 2 years after PAO. Predictors for hip arthroscopy were mild hip dysplasia, cross-over sign and a detached labrum evaluated on MRA. At follow-up 2 years after PAO, the clinical outcome in the non-arthroscopic group is superior to that in the arthroscopy group with statistically significant differences in patient reported outcome measures. In the majority of patients, a PAO without subsequent intraarticular assessment resulted in joint preservation with excellent clinical outcome, and currently we recommend PAO as first choice in patients with hip dysplasia. However, patient with mild dysplasia, cross over sign and detached labrum is at particular risk for the need of a hip arthroscopy and future studies must clarify treatment strategies for this patient group. We agree with Parvizi et al. [10] that hip arthroscopy alone without addressing the bony abnormalities in DDH is in general not recommended since a high reoperation rate has been observed.

The poor results in the arthroscopy group might be explained by the fact that the problem does not come from the hip joint itself, and that the need for hip arthroscopy after PAO might be lower. As a consequence of the present study, we do not refer our patients directly to arthroscopy if the present with pain at follow-up. Instead we focus on extra-articular reasons for pain. Our regimen at follow-up has changed in direction to ultrasound examination in order to address soft tissues around the hip, and we also refer the patients to iliopsoas exercises [36], since we often observe weakness and inflammation of iliopsoas. Currently, we are performing an ongoing prospective study focusing on soft tissue around the dysplastic hip. Which eventually might result in more knowledge about persisting pain in these patients since arthroscopy does not seem to be the answer.

## Funding

Danish Rheumatism Association (R99-A1830) to C.H.A.

CONFLICT OF INTEREST STATEMENT

None declared.
